# Exploring the Nexus of Energy Burden, Social Capital, and Environmental Quality in Shaping Health in US Counties

**DOI:** 10.3390/ijerph18020620

**Published:** 2021-01-13

**Authors:** Tony G. Reames, Dorothy M. Daley, John C. Pierce

**Affiliations:** 1 School for Environment & Sustainability, University of Michigan, 440 Church St., Ann Arbor, MI 48109, USA; 2 School of Public Affairs & Administration and Environmental Studies Program, University of Kansas, Lawrence, KS 66045, USA; daley@ku.edu; 3 School of Public Affairs & Administration, University of Kansas, Lawrence, KS 66045, USA; jcpierce@ku.edu

**Keywords:** energy burden, social capital, environmental quality, public health, social determinants of health

## Abstract

The United States spends more on health care than any other OECD country, yet the nation’s health is declining. Recent research has identified multiple sources for this decline, including one’s position in social and economic structures, environmental quality, and individual and collective social capital. This paper assesses the primary hypotheses that the health effects of household energy burden, social capital and environmental quality on aggregated community health levels remain while controlling for other determinants. The analysis moves beyond prior research by integrating multiple secondary data sources to assess those effects across US counties. Three indicators of public health are analyzed (premature mortality, self-reported health, and life expectancy). The county-level energy burden is measured by the percent of household income spent on housing energy bills for low- and moderate-income households. In addition to energy burden, social capital, environmental quality and other determinants are included in the analysis. The results produced by multivariate regression models support the primary hypotheses, even while a number of control variables also have a significant effect on health. The paper concludes that public health is associated with a complex nexus of factors, including environmental quality and social capital, and that energy burden needs to be among the considerations.

## 1. Introduction

**Figure 1:**
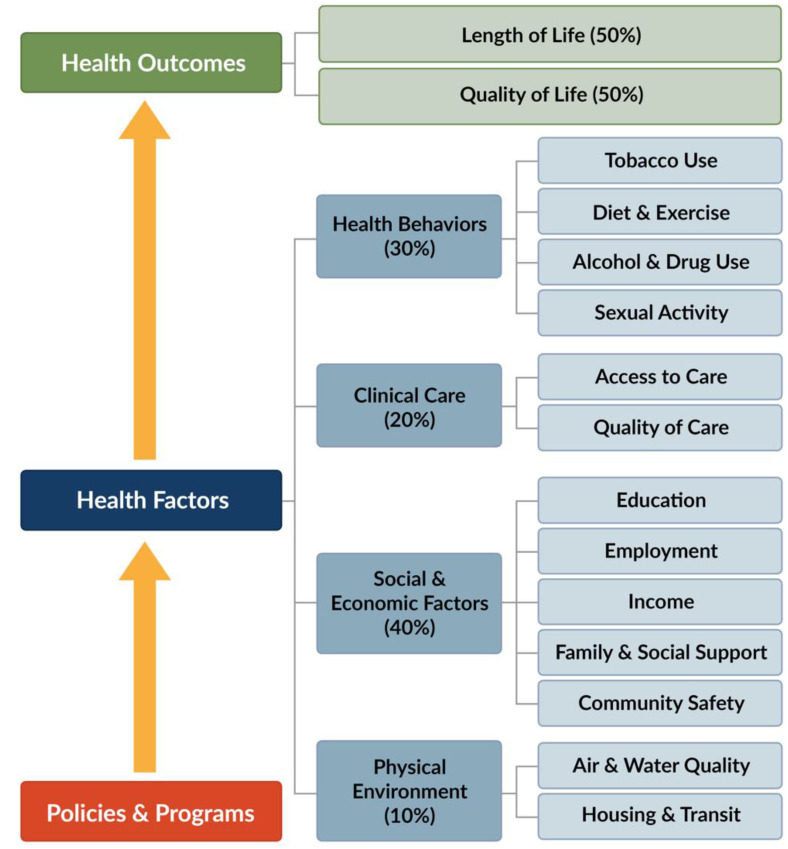
The conceptual framework outlining the social determinants of population health [7]. Country Health Rankings model ©2014 UWPHI.

The United States spends more on health care than any other OECD country. Despite this spending, many health outcomes are moving in the wrong direction. Life expectancy is declining, and chronic diseases, suicide rates, and other negative health outcomes are increasing [1]. Researchers and practitioners alike acknowledge the multitude of factors that determine health [2,3,4]. While access to and quality of health care is important, particularly if someone is ill, broader social, economic, and environmental factors also combine in ways to profoundly shape health and well-being across the life course [5,6]. This paper relies upon insights from the Social Determinants of Health (SDoH) framework as a context within which to better understand how a range of structural factors influence public health in US Counties. Figure 1 outlines the contours of this approach to understanding health. In this conceptual framework, health behaviors and clinical care contribute to public health, but notably, social, economic, and environmental factors also are important explanations for health outcomes across populations.

While there has been considerable research conducted in order to better understand the social determinants of health, more work is needed to further identify how multiple, overlapping determinants may shape that health. This paper examines three different health outcomes in US counties: age-adjusted premature mortality, self-reported health, and life expectancy. Comparing patterns of determinants across these three outcomes helps to identify consistent and critical factors that shape public health. In particular, in addition to social determinants, this research examines the impact of energy burden, social capital, and environmental quality on all three health outcomes.

### 1.1. Energy Burden and Health

Prior research underscores the important relationship between wealth (to which energy burden is linked)—or lack thereof—and health [8,9,10]. A growing body of research suggests that access to affordable household energy is essential for maintaining good health [11,12]. However, energy poverty (that is, insufficient wealth to provide adequate access to energy) is a distinct challenge that threatens a household’s ability to adequately maintain those energy services. The US Energy Information Administration estimates that one in three US households experience some form of energy poverty [13]. Similarly, it also is important to further understand how this particular type of relative resource availability is connected to public health. Energy burden is one measure of energy poverty and a potentially important addition to the determinants of public health [14,15,16,17]. Energy burden reflects household expenditure on energy utilities relative to the household’s gross income capacity [18]. Disproportionate distributions of energy burden (both positive and negative) are evident in particular positions in social and economic systems, such as wealth, education, race or ethnic origin.

Recent research has shown that low income households and households of color spend less on energy overall, yet, they spend a higher proportion of income on energy, and they also spend more on energy per square foot of their domicile [19]. Relative to the concern of this paper, families that have trouble paying their energy bills may sacrifice nutrition, medicine, and other necessities in order to avoid shutoff. More than 25 million US households reduce or forgo food or medicine in order to pay energy costs [13]. Additionally, nearly 13 million US households experience leaving their homes at unhealthy temperatures [13]. Living in underheated homes puts adolescents at double the risk of respiratory problems and at five times the risk of mental health problems [20]. Furthermore, “…living in homes that are not properly heated or cooled increases cases of asthma, respiratory problems, heart disease, arthritis, and rheumatism” [13,21,22,23,24]. Analyzing how energy burden, as an economic stressor, impacts health is important in that it can inform policy interventions that may improve public health.

### 1.2. Social Capital and Health

Social capital—the individual and collective resource that emanates from trust and reciprocity-based networks—is one of the most frequently identified sources of variation in public health [25]. Indeed, community social capital is an established and important determinant of health and well-being [25,26,27,28,29,30,31,32,33,34,35]. Social capital has been shown to have broad-based impacts on public health levels even in the context of other forces that effect health, namely economic stress and socio-demographic variables, such as income and education [36]. The networks providing social capital offer mutual support, opportunities for collaboration and an avenue for health-related activities and information that can enhance well-being. Higher levels of social capital are consistently linked to positive health outcomes; this relationship holds hold across a range of health outcomes regardless of how social capital is measured [37,38,39,40,41,42,43].

### 1.3. Environmental Quality and Health

Past research indicates that environmental quality also is linked to health. Decades of research have firmly established that environmental quality is a consistent determinant of health and that environmental quality is a major concern for both public health officials and the general public in the US. [44,45,46]. An extensive body of research has demonstrated the adverse health outcomes associated with poor environmental quality (in particular, air pollution exposure, specifically PM_2.5_ or particulate matter ≤ 2.5 in aerodynamic diameter) is an important predictor of health levels [47,48,49,50,51,52]. Epidemiological evidence shows air pollution effects on neuropsychological development and impairment as well as on cognitive deficits and behavioral impairment in children and the elderly [50]. Some populations are at greater risk of mortality from the effects of poor environmental quality. For instance, older individuals with comorbidities such as myocardial infarction or diabetes are at greater risk of death associated with high exposure to PM_2.5_ [47]. The risk of hospital admission and death from cardiovascular causes increase significantly with increased concentrations of PM_2.5_ [48,49,51,52]. Moreover, increasing evidence suggests racial/ethnic minorities and low socioeconomic status populations experience greater exposure to PM_2.5_, which may contribute to racial/ethnic and socioeconomic disparities in the adverse health outcomes associated with air pollution exposure [53,54,55].

### 1.4. SDoH Control Variables

The SDoH conceptual framework suggests that there are several other critical drivers of health over and above the three described earlier [5]. While these other factors are not the main focus of the research reported in this paper, they are nonetheless important to consider. Thus, income inequality, housing quality, food insecurity, educational attainment, and access to health care all have been shown to contribute to health outcomes [2,4,9,56]. In the US there are large and persistent racial disparities in health [57,58]. Discrimination and structural and cultural racism remain a fundamental cause shaping population health [58,59,60].

### 1.5. Expectations

Of the three variables of interest, energy burden is the least studied for its relationship with public health, particularly in the US context. In order to fully understand how energy burden connects to health, it is necessary to control for important competing explanations of health. This study moves beyond previous research by placing the effects of energy burden empirically within the context of the SDoH framework, by expanding the empirical setting to more than 2000 counties in the US (not only the larger cities subset most frequently studied), and by considering multiple measures of public health outcomes in US counties. Understanding the impact of energy burden on health outcomes is important. Given the complex nature of health, the critical question remains: does energy burden affect public health outcomes over and above the independent influence of social capital, environmental quality and other social determinants of health? 

In order to focus this research, the present study of energy burdens, social capital, environmental quality and public health engages the following hypotheses:

**Hypothesis** **1** **(H1).** Higher levels of energy burden within a county will be associated with poorer health outcomes, even when controlling for social capital, environmental quality, and a range of important social determinants of health.

**Hypothesis** **2** **(H2).** Higher levels of social capital within a county will be associated with better health outcomes, even when controlling for energy burden, environmental quality, and a range of established social determinants of health.

**Hypothesis** **3** **(H3).** Poorer environmental quality will be associated with poorer health outcomes within a county, even when controlling for energy burden, social capital, and a range of established social determinants of health.

## 2. Materials and Methods 

A range of existing county-level secondary data sources are employed here in order to better understand the complex structural determinants of public health. Multiple data sets are merged using County FIPS codes. The present research begins by collecting information on three different health outcomes across all US counties. The analysis relies on the County Health Rankings and Roadmap (CHRR) project for the measures of health and many of the variables noted in the SDoH framework. The CHRR data are augmented with other county-level secondary data sets reporting social capital and energy burden. 

### 2.1. Data and Variables 

Table 1 describes the variables used in this analysis. Health is a multifaceted concept not easily captured in a single empirical measure. Therefore, three different measures of health are employed in the models as separate dependent variables: premature mortality; self-reported health; and life expectancy. Premature mortality is a widely used indicator of population health. This is an age-adjusted variable where deaths that occur at younger ages are weighed more in the measure. Thus, premature mortality reports the number of deaths of county residents who are under 75 years. To compare across counties, this information is normalized by population and averaged across three years (2016–2018). In addition to premature mortality, the models used here also consider the percent of residents in a county who report fair or poor health. These data are found in the CHRR project and are drawn from the Center for Disease Control and Prevention’s Behavioral Risk Factor Surveillance System. Self-reported health also is a widely employed indicator of health [61,62,63]. The final dependent variable is life expectancy, also reported in the CHRR project. This information is drawn from the National Center for Health Statistics and is an age-adjusted measure reporting the average life expectancy in a county. While this research presents a cross-sectional analysis, the variables representing health outcomes are based on data collected between 2016 and 2018 (as noted in Table 1). To strengthen the research design, the data representing the independent variables are based on information collected that predates the health outcomes examined in this research.

Most of the independent variables employed here also are drawn from the CHRR project. However, measures of energy burden originate from the US Department of Energy (DOE). The Low-Income Energy Affordability Data (LEAD) Tool, created by the DOE, presents data, maps and graphs for understanding housing and energy characteristics for low- and moderate-income (LMI) households. From the LEAD Tool, the average county-level energy burden variable is calculated for electricity, natural gas, and other fuel expenditures. The energy burden variable is the percentage of income spent on housing energy bills for LMI households, where LMI is defined as households earning between 0 and 80% of the Area Median Income (AMI). Energy burden data from the LEAD Tool have been used to explore the spatial distribution of energy vulnerability across the US and correlations with mortality rates and various demographic and socioeconomic characteristics at the county level [65].

The social capital measure used here is based on previously published and archived data [64]. This index score is produced by a principal component analysis of four county-level variables: including per capita civic associations, non-profit organizations, voter turnout and census participation. This measure has been widely used and is considered a valid measure of county social capital [66]. The analysis also includes a measure of environmental quality; relying upon the CHRR project, it includes a measure of air quality, specifically the average level of PM_2.5_ in a county in 2014.

Using the SDoH framework as a guide, the analysis includes several control variables, all of which are extracted from the CHRR project. These measures include income inequality – a ratio of household income at the 80th percentile in the county compared to household income at the 20th percentile in the county [57]. Inadequate housing measures the percentage of households in a county that either experience over-crowding or inadequate plumbing. In the US, there are persistent racial disparities in health, therefore the analysis includes a measure of the percent of residents who identify as Non-Hispanic Black [58]. Access to healthy food and access to health care providers are also included as control variables and are found in the CHRR data set. The final control variable is educational attainment in the form of the percentage of adults with some post-secondary education. 

These data are merged using County FIPS identifiers to construct a unique secondary data set that can examine the relative influence of energy burden, social capital, and environmental quality while controlling for other important determinants of health. Descriptive statistics are included in Appendix A.

### 2.2. Methods

This analysis explores how county-level factors shape health outcomes. In the US, counties are embedded within states and thus differences across states are likely to impact health. Therefore, this analysis uses a state fixed effect approach to model premature mortality, self-reported health and life expectancy. Preliminary diagnostics revealed spatial patterning in all three models making ordinary least squares (OLS) regression analysis inappropriate. Global Moran’s I coefficient and its statistical significance were computed on model residuals to identify spatial autocorrelation [67]. For all three models, tests revealed a Moran’s I, *p* < 0.001, indicating that model variables are in some way spatially clustered. Given such distributions, simple regression models would not account for spatially correlated errors and model results are likely to biased. Therefore, this analysis uses spatial error regression models to provide the most robust parameter estimates.

The choice of a spatial error models (SEM), as opposed to a spatial lag approach, is based both on statistical and theoretical grounds [68]. SEM assumes that the explanatory variables alone do not account for the spatial autocorrelation. This analysis relies on county level aggregate data and as such, we are not able to account for individual health behaviors that are part of the SDoH conceptual framework. These omitted parameters are likely to have spatially correlated factors, making a SEM suitable.

The SEM takes the following form:

(1) $$y=\alpha \;\;+\;\;\sum_{k}\beta_{k}X_{k\;}\;+\;\lambda We+u$$

where y represents one of the three dependent variables (premature mortality, self-reported health or life expectancy), α is the constant, *β* is the coefficient for the *k* number of independent variables, λ is the spatial autoregressive coefficient, and *W* is the spatial weighting matrix, e is the random error term from OLS regression, and u is the spatially independent error term.

As the primary interest of this study is to understand how explanatory variables shape health outcomes, we rely on contiguity-based spatial weights. Contiguity-based spatial weights were estimated in Stata 16 using polygon map files from the US Census Bureau.

## 3. Results

Table 2, Table 3 and Table 4 present the results of the three analyses for factors shaping premature mortality, self-reported health, and life expectancy in US counties, respectively. The OLS model results are also presented for reference; the results of the independent variables are nearly the same. The SEM results are discussed hereafter. All three models highlight a consistent—and significant—relationship between energy burden and health. Communities that have more LMI households experiencing higher energy burdens also have poorer health outcomes. As energy burden increases so too do premature mortality rates within a county (Table 2). Across US counties, each unit of increase in LMI energy burden is associated with an average 240 more premature deaths per 100,000 people between 2016 and 2018. Similarly, as the energy burden increases across counties, each unit increase is associated with a seven percent increase in county residents that report experiencing fair or poor health (Table 3). Finally, each unit increase in energy burden is significantly associated with more than a five year decrease in county average life expectancy (Table 4).

As noted earlier, prior research has established that social capital is an important determinant of health [25,28,29]. The results here are consistent with that past research. Social capital is statistically significant in the models of self-reported health and life expectancy. Higher levels of social capital are systematically related to lower percentages of residents reporting fair or poor health (Table 3). A county with a 10-point higher social capital score relative to another county experiences roughly 4 percent fewer residents reporting fair or poor health. Counties with higher levels of social capital also have significantly higher levels of life expectancy (Table 4). Each 10-point increase in social capital was associated with an increased average life expectancy of 2 years.

The measure of environmental quality (the annual average level of PM_2.5_ in a county in 2014) is significant in only one of the three models, and the result is as expected. Changes in environmental quality are no more or less associated with rates of premature mortality or self-reported health across counties. Higher levels of PM_2.5_ are associated with statistically significant lower rates of life expectancy (Table 4). Each 10-point increase in PM_2.5_ across US counites is associated with a two year decrease in average life expectancy.

Overall, the remaining control variables suggest strong support for the SDoH framework. Income inequality—measured here as the ratio of household income at the 80th percentile to household income at the 20th percentile—is systematically related to poor health outcomes. This is in keeping with previous research findings; growing income inequality is significantly associated with all three measures of health [69,70,71,72]. Higher income inequality is linked to higher rates of premature mortality. Similarly, counties where the gap between the 80th and 20th percentile of household income is high also have systematically higher percentages of residents reporting fair or poor health. Finally, higher levels of income inequality within a county are linked to lower levels of life expectancy. 

Inadequate housing (measured as the percentage of households that are overcrowded or lack plumbing or kitchen facilities) is significant in predicting increased premature mortality and self-reported poor health. In keeping with the large and growing literature that documents racial health disparities, the results indicate that race matters in shaping health. Counties that have a higher percentage of residents identifying as Non-Hispanic Black also have statistically higher rates of premature mortality, a higher percentage of residents reporting fair or poor health, and lower life expectancy. Similarly, access to healthy food is an important predictor of all three measures of health. Recall that this is the percentage of low-income residents who do not have access to a grocery store. As this percentage increases across counties, there are corresponding increases in rates of premature morality, increases in percentages of residents who report fair or poor health and declining rates of life expectancy.

The results also suggest that access to a physician matters, but not for all measures of health. In particular, this control variable is significant in only one of the models, namely premature mortality (Table 2). More per capita access to physicians is associated with lower rates of premature mortality in a county. The final control variable, education, is statistically significant across all three models and the results are consistent with prior research on the link between education and health. Counties that have more educated residents have lower rates of premature mortality, lower percentages of residents reporting fair or poor health, and higher life expectancy.

## 4. Discussion

Modeling three different measures of health provides an opportunity to compare more fully the relationships between health on the one hand, and energy burden, social capital, and environmental quality on the other hand. A challenge of this study was controlling for multiple determinants of health; nonetheless, strong patterns emerged across all three measures of health. Finding patterns of significance across models explaining different measures of health surely increases confidence in the results. Indeed, most of the variables had similar and expected relationships with health measures across the three models.

A limited number of studies have empirically explored the relationship between energy burden and health in the U.S while controlling for other variables known to have either positive or negative effects on public health. It was anticipated that higher energy burdens would be associated with poorer health outcomes. Across nearly all US counties, the analysis supports this expectation. Moreover, modeling results suggest this is an influential determinant of health across all three models, with only education and race having stronger influences on the health outcomes. Energy poverty is thus an important addition to the broader SDoH framework. High energy burdens for LMI households are particularly detrimental for population health. For instance, in the US, recipient families of the Low-Income Home Energy Assistance Program (LIHEAP) which provides financial assistance with energy bills, report often choosing between paying their energy bill or buying food, a situation commonly referred to as “heat or eat” which poses high health risks of malnutrition for children [73]. Recall also that the US Energy Information Administration also found that more that 25 million households reduce or forgo food or medicine in order to pay their energy bills [13]. If energy burdened households are unable to afford medical treatments, it is to be expected that the communities in which they live would experience increased levels of premature mortality, reduced life expectancy, and higher percentages of the population reporting they are in poorer health. Many state and local governments are beginning to consider the health implications of energy unaffordability and are launching programs that focus on improving energy efficiency and/or access to renewable energy in order to reduce energy consumption, improve housing quality, and reduce energy bills.

Social capital exhibits a significant positive effect on two of the three health measures, even when controlling for the effects of energy burden, environmental quality and other social determinants. Thus, to some degree it may be possible for the trust-based networks to compensate some for the negative health effects of energy burden. If greater energy burden produces an environment that either directly or indirectly is likely to lead to lower public health levels, the collective resource of social capital may produce some counterweight to energy burden’s negative health influence. The trust and reciprocity embedded in social capital’s foundation may be likely to spill over into some reservoir of support. However, it is important to note that social capital—while durable in some settings—is nonetheless dynamic and if depleted or low, is likely to take considerable time to develop. As social capital varies across counties, the resources associated with that capital also vary. A strategy to advance health by investing in social capital is a longer-term investment in community health and well-being.

Surprisingly, after accounting for spatial clustering, environmental quality was statistically associated with only one measure of public health, life expectancy. But measures of air pollution also can introduce complications. In this study, a single, annual average value of PM_2.5_ at the county level is used; as a result, variability in air quality across a county is not well accounted for. Particulates may be directly emitted from a source such as engine exhaust fumes or formed in the atmosphere as a result of chemical reactions such as industrial activity. Therefore, PM2.5 tends to be higher in more urban areas with a higher level of traffic. Thus, the association between air pollution and health outcomes may need to be assessed at a smaller spatial scale than the county-level, such as zip code or census tract. However, other studies have found that higher PM_2.5_ exposure was not associated with perceptions of higher concern about pollution-related health risks [74].

To be sure, this study is constrained by its character as an aggregate, observational analysis that does not directly observe the pathways through which energy burdens operate to shape health outcomes. However, other more limited previous observational studies suggest that theses pathways are present and provided the foundation for the hypotheses guiding this aggregate level study. The explicit analysis of pathways across large numbers of counties is an area for future study. It is also important to note that this study is confined to a single country, the US. While the nation-wide county-level database used here provides substantial variation in the size and cultures of the energy burden context, at the same time these findings may or may not be replicated in other national contexts. Results in other countries may vary based on different conjunctions of energy burden and health, as well as energy economies that are supported by different patterns of wealth, energy governance, or by energy sources that impinge on public health to a significantly different degree.

## 5. Conclusions

This study supports the social determinates of health framework and suggests that energy poverty should be included as a central component. To better understand how multiple, overlapping social determinants shape health, this study examined three different health outcomes across the majority of US counties: age-adjusted premature mortality, self-reported health, and life expectancy. In particular, this research examined the impact of energy burden, social capital, and environmental quality and their influence on all three health outcomes. 

The research reported here clearly leads to the conclusion that the aggregate cost and availability of energy relative to the wealth capacity of individuals to pay for it has a significant effect on the health of those individuals. Those health effects of energy burden maintain across a range of health measures, from self-assessment to life expectancy to premature mortality. Moreover, those independent effects of energy burden emerge even when controlling for the well-established effects of social capital, environmental quality, and a broader set of social determinants of health. However, it also is clear that identifying the health effects of energy burdens does not erase the health effects of social capital and the social determinants of health more broadly. Thus, this energy burden analysis enhances both the understanding of the complexity of the causes of public health when aggregated at the county level and expands knowledge in a way that should provide new and innovative pathways through which public health can be enhanced, or at least can be protected. The implications of this paper contain a dynamic that may travel beyond the boundaries of health or energy burdens themselves. Additional concerns with energy justice may reside in a location external to energy burdens when those burdens themselves are disproportionately distributed among vulnerable populations, or when the negative health effects of those burdens are likewise inequitably distributed. If so, energy burden mitigation can provide a separate pathway toward the goal of public health equity.

## Figures and Tables

**Table 1:** Description of variables.

Variable	Description
Premature Mortality	This is the age-adjusted measure of premature mortality, the number of deaths among residents in a county who are under the age of 75 per 100,000 population. Reported in County Health Rankings and Roadmap (CHRR) using data from the National Center for Health Statistics from 2016–2018.
Self-Rated Health	The percentage of adults, age adjusted, within a county reporting fair or poor health. This is estimated using representative population health data (the Centers for Disease Control and Prevention’s (CDC’s) Behavioral Risk Factor Surveillance System) collected in 2017.
Life Expectancy	This is an age-adjusted measure that reports the average number of years a person can expect to live. Life expectancy accounts for the number of deaths in a given time period and the number of people at risk of dying during that time period. Reported in CHRR using data from the National Center for Health Statistics from 2016 to 2018.
Energy Burden	The county-level average proportion of income spent on housing energy bills for low- and moderate-income households. This measure is calculated using county-level Low-Income Energy Affordability Data available from the US Department of Energy. This was reported in 2016.
Social Capital	An index score compiled from publicly available sources and updated in 2014 [64]. This is based on a principal component analysis of four county-level variables: (1) the aggregate number of associations per capita including civic association, bowling centers, public golf courses, fitness centers, sports, religious, political, labor, business, and professional organizations per 10,000 people; (2) non-profit organizations without an international focus; (3) voter turnout, and (4) 2000 census response rate.
Environmental Quality	Average level of PM_2.5_ in a county in 2014. Reported in the CHHR using data from the CDC’s Environmental Public Health Tracking Network.
Income Inequality	Using 5-year estimates, this is the ratio of household income at the 80th percentile to the income at the 20th percentile. Reported in CHHR using data from the American Community survey from 2014 to 2018.
Inadequate Housing	The percentage of households within a county that are overcrowded or lack kitchen or plumbing facilities. Reported in CHHR using data from the American Community survey from 2014 to 2018.
Non-Hispanic Black	The percent of non-Hispanic Black or African American residents in a county in 2014. Compiled from Census data and available via the CHRR program.
Healthy Food Access	The percentage of low-income residents who do not live close to a grocery store in 2015. These data are compiled from USDA Food Atlas and available via the CHRR.
Access to Physicians	The ratio of primary care providers to the population in the county (per 100,000 people). These data are compiled by the American Medical Association and available via the CHRR.
Education	The percentage of adults in a county that are age 25–44 with some post-secondary education. Reported in CHHR using data from the American Community survey from 2014–2018.

**Table 2:** The results of spatial error models of factors shaping premature mortality in US counties.

Premature Mortality	OLS Model				Spatial Error Model			
	Coefficient	Std Error	95 % CI		Coefficient	Std Error	95 % CI	
Energy Burden	267.58	(25.42) ***	217.73	317.42	239.63	(26.45) ***	187.79	291.48
Social Capital	−3.28	(1.55) *	−6.31	−0.25	−2.10	(1.53)	−5.10	0.90
Environmental Quality	0.30	(1.43)	−2.50	3.11	−0.06	(1.65)	−3.30	3.17
Income Inequality	28.36	(2.36) ***	23.72	33.00	24.37	(2.27) ***	19.92	28.83
Inadequate Housing	99.70	(78.17)	−53.58	252.99	293.91	(77.68) ***	141.65	446.16
Non-Hispanic Black	71.31	(14.91) ***	42.08	100.54	102.38	(16.48) ***	70.08	134.67
Healthy Food Access	172.15	(22.34) ***	128.34	215.96	163.53	(21.54) ***	121.31	205.75
Access to Physicians	−15,557.55	(4773.23) **	−24,916.94	−6198.15	−16,171.95	(4485.64) ***	24,963.65	7380.25
Education	−277.47	(17.33) ***	−311.46	−243.48	−266.10	(16.86) ***	−299.15	−233.04
Constant	423.13	(23.07) ***	377.90	468.35	428.31	(25.85) ***	377.64	478.97
Lambda, λ					0.48	(0.03) ***	0.42	0.54
*n*	2871				2871			
R^2^	0.60							
Adjusted R^2^	0.59							
pseudo R^2^					0.60			

Standard errors in parentheses; state fixed effects not shown. * *p* < 0.05, ** *p* < 0.01, *** *p* < 0.001.

**Table 3:** The results of spatial error models of factors shaping self-reported health in US counties.

Self-Reported Health	OLS Model				Spatial Error Model			
	Coefficient	Std Error	95 % CI		Coefficient	Std Error	95 % CI	
Energy Burden	7.65	(0.66) ***	6.27	8.87	7.39	(0.68) ***	6.06	8.73
Social Capital	−0.42	(0.04) ***	−0.54	−0.38	−0.42	(0.04) ***	−0.50	−0.35
Environmental Quality	−0.11	(0.04) **	−0.20	−0.05	−0.05	(0.04)	−0.14	0.03
Income Inequality	1.15	(0.06) ***	1.08	1.32	0.99	(0.06) ***	0.88	1.11
Inadequate Housing	37.03	(2.00) ***	33.72	41.70	34.31	(1.94) ***	30.50	38.11
Non-Hispanic Black	8.57	(0.39) ***	7.74	9.27	9.79	(0.43) ***	8.94	10.64
Healthy Food Access	4.41	(0.52) ***	3.69	5.97	3.55	(0.49) ***	2.59	4.51
Access to Physicians	−117.50	(119.80)	−498.17	−2.05	−133.27	(109.09)	−347.09	80.55
Education	−11.00	(0.43) ***	−11.60	−9.83	−10.25	(0.41) ***	−11.05	−9.45
Constant	19.00	(0.60) ***	17.63	20.00	18.71	(0.69) ***	17.37	20.06
Lambda, λ					0.58	(0.03) ***	0.53	0.64
*n*	2925				2925			
R^2^	0.84							
Adjusted R^2^	0.83							
pseudo R^2^					0.84			

Standard errors in parentheses; state fixed effects not shown. ** *p* < 0.01, *** *p* < 0.001.

**Table 4:** The results of spatial error models of factors shaping life expectancy in US counties.

Life Expectancy	OLS Model				Spatial Error Model			
	Coefficient	Std Error	95 % CI		Coefficient	Std Error	95 % CI	
Energy Burden	−6.32	(0.71) ***	−7.72	−4.92	−5.63	(0.75) ***	−7.09	−4.17
Social Capital	0.23	(0.04) ***	0.13	0.31	0.21	(0.04) ***	0.12	0.29
Environmental Quality	−0.16	(0.04) ***	−0.24	−0.08	−0.19	(0.05) ***	−0.29	−0.10
Income Inequality	−0.63	(0.07) ***	−0.76	−0.50	−0.56	(0.06) ***	−0.68	−0.43
Inadequate Housing	4.07	(2.20)	0–0.24	8.40	−0.63	(2.19)	−4.93	3.66
Non-Hispanic Black	−1.23	(0.42) **	−2.06	−0.41	−1.88	(0.46) ***	−2.79	−0.97
Healthy Food Access	−2.58	(0.66) ***	−3.86	−1.29	−2.45	(0.63) ***	−3.68	−1.21
Access to Physicians	133.52	(134.45)	−130.10	397.15	114.98	(126.61)	−133.17	363.14
Education	8.05	(0.49) ***	7.08	9.01	7.87	(0.48) ***	6.94	8.81
Constant	77.47	(0.65) ***	76.19	78.74	77.85	(0.73) ***	76.42	79.28
Lambda, λ					0.48	(0.03) ***	0.42	0.54
*n*	2859				2859			
R^2^	0.54							
Adjusted R^2^	0.54							
pseudo R^2^					0.55			

Standard errors in parentheses; state fixed effects not shown. ** *p* < 0.01, *** *p* < 0.001

## Appendix A

**Table A1:** Descriptive Statistics.

	Mean	Std Dev	Min	Max
Premature Mortality	407.05	111.18	127.77	1216.80
Self-Reported Health	17.94	4.65	8.12	40.99
Life Expectancy	77.43	2.92	61.63	104.74
Energy Burden	0.13	0.09	0.02	0.67
Social Capital	−0.05	1.17	−3.18	21.81
Environmental Quality	9.15	1.90	3.00	19.70
Income Inequality	4.52	0.74	2.54	11.97
Inadequate Housing	0.03	0.02	0.00	0.38
Non-Hispanic Black	0.09	0.14	0.00	0.85
Healthy Food Access	0.08	0.06	0.00	0.72
Access to Physicians	0.00	0.00	0.00	0.01
Education	0.58	0.11	0.20	0.90
*n*	2853			
